# An interpretable machine learning model based on a quick pre-screening system enables accurate deterioration risk prediction for COVID-19

**DOI:** 10.1038/s41598-021-02370-4

**Published:** 2021-11-30

**Authors:** Lijing Jia, Zijian Wei, Heng Zhang, Jiaming Wang, Ruiqi Jia, Manhong Zhou, Xueyan Li, Hankun Zhang, Xuedong Chen, Zheyuan Yu, Zhaohong Wang, Xiucheng Li, Tingting Li, Xiangge Liu, Pei Liu, Wei Chen, Jing Li, Kunlun He

**Affiliations:** 1 Department of Emergency, The First Medical Center to Chinese People’s Liberation Army General Hospital, Beijing, China; 2 Washington University in St. Louis, St. Louis, USA; 3 School of Economics and Management, Beijing Jiaotong University, Beijing, China; 4 Department of Emergency, Affiliated Hospital of Zunyi Medical University, Zunyi, China; 5 School of Management, Beijing Union University, Beijing, China; 6 School of E-Business and Logistics, Beijing Technology and Business University, Beijing, China; 7 Department of Emergency, The Third Medical Center to Chinese People’s Liberation Army General Hospital, Beijing, China

**Keywords:** Infectious diseases, Risk factors, Machine learning, Predictive medicine

## Abstract

A high-performing interpretable model is proposed to predict the risk of deterioration in coronavirus disease 2019 (COVID-19) patients. The model was developed using a cohort of 3028 patients diagnosed with COVID-19 and exhibiting common clinical symptoms that were internally verified (AUC 0.8517, 95% CI 0.8433, 0.8601). A total of 15 high risk factors for deterioration and their approximate warning ranges were identified. This included prothrombin time (PT), prothrombin activity, lactate dehydrogenase, international normalized ratio, heart rate, body-mass index (BMI), D-dimer, creatine kinase, hematocrit, urine specific gravity, magnesium, globulin, activated partial thromboplastin time, lymphocyte count (L%), and platelet count. Four of these indicators (PT, heart rate, BMI, HCT) and comorbidities were selected for a streamlined combination of indicators to produce faster results. The resulting model showed good predictive performance (AUC 0.7941 95% CI 0.7926, 0.8151). A website for quick pre-screening online was also developed as part of the study.

## Introduction

In early December 2019, Wuhan, Hubei Province, China, emerged as the epicenter of an unfamiliar pneumonia. On January 3, 2020, Chinese scientists had isolated the severe acute respiratory syndrome coronavirus 2 (SARS-CoV-2; previously called 2019-nCoV) in samples of bronchoalveolar lavage fluid from an infected patient^1^. On February 11, 2020, the World Health Organization (WHO) designated this disease as coronavirus disease 2019 (COVID-19). The WHO later reported 8,096 SARS cases and 774 deaths across 29 countries, suggesting an overall case fatality rate (CFR) of 9.6%. In addition, MERS was still not fully controlled with ~ 2494 confirmed cases and 858 deaths across 27 countries, yielding a CFR of 34.4%. Despite much higher CFR values for SARS and MERS, COVID-19 has led to more total deaths due to a relatively high contagiousness and lack of an effective vaccine or drug^2–4^. As of 30 November 2020, COVID-19 had quickly spread to a majority of countries worldwide, causing nearly 1,456,687 deaths^5^. Although roughly 81% of COVID-19 patients exhibit mild or moderate symptoms, some have been observed to deteriorate suddenly, rapidly developing into the severe or critically ill categories^6,7^. As such, identifying the early warning indicators of critical illness is of significant importance. If such signs could be recognized early in the treatment process, patients could be allocated increased attention, thereby reducing mortality. However, the majority of published studies on the adverse prognosis of COVID-19 have used statistical methods to describe both the characteristics and outcomes of COVID-19 patients, by comparing severe and non-severe patients to identify risk factors^8–15^. However, this approach does not provide an early prediction of a poor prognosis. In studies using machine learning algorithms to predict prognoses for COVID-19 patients, prediction outcomes have mostly been limited to intensive care unit (ICU) admissions and death^16–18^. In contrast, we define the transition to deterioration as the predicted outcome. Although previous studies have achieved good predictions, the number of indicators required for is typically large and complex, including a variety of laboratory indicators^16–18^. This can lead to long wait times for acquiring indicators, ignoring the problem of accessibility when using machine learning models in practical scenarios. In addition, studies based on traditional statistical models or machine learning algorithms have mostly identified risk factors for patient deterioration or in-hospital mortality, but typically do not provide corresponding early warning ranges. With these needs in mind, this study used machine learning to predict the deterioration of COVID-19 patients, identifying risk factors and approximate early warning ranges. By focusing on applicability and practicality, we have reduced the number of indicators required by the model and the corresponding wait time. This allowed for predictions using only five indicators, of which only two were assays, for quick bedside testing. This could result in guided interventions and improve the overall quality of care.

The primary outcomes of this study are as follows. (1) An interpretable machine learning algorithm was used to construct an accurate and effective model for predicting whether mild/moderate patients would deteriorate into severe/critical cases. Two combined stepped indices were formed based on the varying quantities required by the model. (2) Risk factors were also identified, and the corresponding approximate warning ranges (for severe COVID-19) were represented using Shapley additive explanations plots. (3) The results were integrated into a website serving as an online pre-screening tool.

## Methods

### Patient and public involvement

This was a retrospective case series study in which no patients were involved in designing the study, developing research questions, or measuring outcomes. In addition, no patients were asked to advise in the interpretation or dissemination of results.

### Patient data and study design

This retrospective, single center study recruited patients from Feb 2 to Apr 1, 2020, at Huoshenshan Hospital in Wuhan, China. All study patients were diagnosed as having COVID-19 pneumonia by a positive result from a nucleic acid test and were divided into 4 clinical classifications (mild, moderate, severe, and critically ill) using the diagnosis and treatment protocol for novel coronavirus pneumonia (6th edition). These criteria are maintained by the National Health Commission of the People’s Republic of China (see Additional file 1). In this study, patients who were either mild or moderate were treated as mild cases. All other patients were considered severe. The primary goal of the study was predicting whether patients would deteriorate from mild to severe status. Thus, we used longitudinal data derivates from patients whose initial status was mild but subsequently deteriorated. Specifically, 1537 of the 3028 patients had at least one status marked as severe, and 1140 of the 1537 patients experienced deterioration (other patients only experienced a transition from severe to mild or remained severe). We analyzed the time series of these 1140 patients. For each patient and at each time point, if the status changed from mild to severe, the time series data up to this point was labeled as positive (the experimental group), otherwise, the data was labeled as negative (the control group).

### Data collection and processing

Electronic medical records (EMR) were collected from all patients at Huoshenshan Hospital during admission, including epidemiological, demographic, clinical, laboratory, medical history, exposure history, comorbidities, symptoms, chest computed tomography (CT) scans, and any treatment measures (i.e., antiviral therapy, corticosteroid therapy, respiratory support, and kidney replacement therapy). All data were reviewed by a trained team of physicians. To more accurately identify the high-risk factors that cause mild patients to deteriorate into severe/critical patients, mild patients were divided into severe (the experimental group) and non-severe (the control group) categories based on whether they deteriorated into severe cases during hospitalization (see Fig. 10A). However, disease progression was dynamic and 35.7% of patients in the severe group experienced more than one deterioration event during hospitalization (an average of 2.9 times per patient). Each of these transitions was considered a positive sample in the study, allowing the model to acquire more information between features. In contrast, patients in the control group were in a constant mild state, providing a sufficient source of negative samples We divided the experimental group and the control group based on the state of patients. Therefore, in 1140 patients that deteriorated from mild to severe, the periods of mild state provided a source of negative samples, which led to class imbalances as the number of negative samples was significantly higher than that of positive samples. As such, a random under-sampling technique was used to establish two classes of equal size^19,20^.

Model input included three broad classes of variables (i.e., features) that are commonly available in EMR: (1) demographic variables (e.g., age and sex); (2) comorbidities; and (3) clinical and laboratory results.

The type of missing data was Missing Completely at Random (MCAR). The probability of an observation being missing depended on the frequency of recording. For instance, a patient may have declined a test, or a doctor may have forgotten to record test results. There was no hidden mechanism related to features and it did not depend on any characteristic of the patients.

Values that were far from the true recorded values were defined as missing values because we did not want to leak distant future information. Specifically, if a feature for a patient was not recorded frequently, the data between distant record points was missing data. Therefore, if a patient had no or only a few records for a feature (missing rate ≥ 50%), we deleted all values for this feature and all the values were missing. The process above was carried out at the patient level, which means each patient’s series was treated this way.

Next, we handled the missing values at the feature level. First, we removed features with missing rate greater than 50%. Then, we applied random forest imputation to fill missing values, which results in a better performance (see Fig. 10B)^21–24^. Overall, this produced 82 features for inclusion in the model (see Additional file 2).

Furthermore, tenfold cross validation was adopted to evaluate model performance. In this process, the dataset was randomly partitioned into 10 equal-sized subsamples, nine of which were used to train the model, which was then validated using the remaining subsamples (see Fig. 10C). Accuracy, recall, precision, F_1_ score, and area under the receiver operating characteristic (AUC) curve were used to assess model performance (see Additional file 3).

### Machine learning algorithms

This study considered interpretability to be a core requirement for machine learning model selection^25,26^. Extreme gradient boosting (XGBoost) and logistic regression (LR) algorithms were used to predict whether a patient with mild COVID-19 symptoms would develop into a severe case. XGBoost, proposed by Chen et al.^27^, has produced unprecedented results for a variety of machine learning problems^25,28–31^. XGBoost works by using the decision tree as a weak classifier for iteratively modifying the residuals of previous models^27^. In addition, the algorithm includes a regularization component to control the complexity of the tree, thereby avoiding overfitting and simplifying the model^27^. Logistic regression (LR), a conventional machine-learning algorithm, has been widely used for classification tasks in medicine^31–37^. Rather than fitting a straight line or hyperplane, a logistic function can be used to constrain the output of a linear equation to between 0 and 1.

Shapley additive explanations (SHAPs) were used to enhance the interpretability of the results^38^. The goal of SHAP is to explain the prediction of an instance *x* by calculating the contribution of each feature to the prediction^39^. Additionally, partial SHAP dependency plots were used to illustrate the effect of individual feature changes on the severity of COVID-19. The SHAP dependence plot represents the marginal effects that each feature has on the predicted outcome of a machine-learning model and could reveal the exact form of this relationship (i.e., linear, monotonic, or more complex)^38^. An additional combined feature effect, after accounting for individual features like the interaction effect, was also considered in this study.

### Ethical approval and consent to participate

The Medical Ethics Committee of the PLA General Hospital approved the study.

### Consent for publication

Not applicable.

## Results

### Patient characteristics

**Figure 1:**
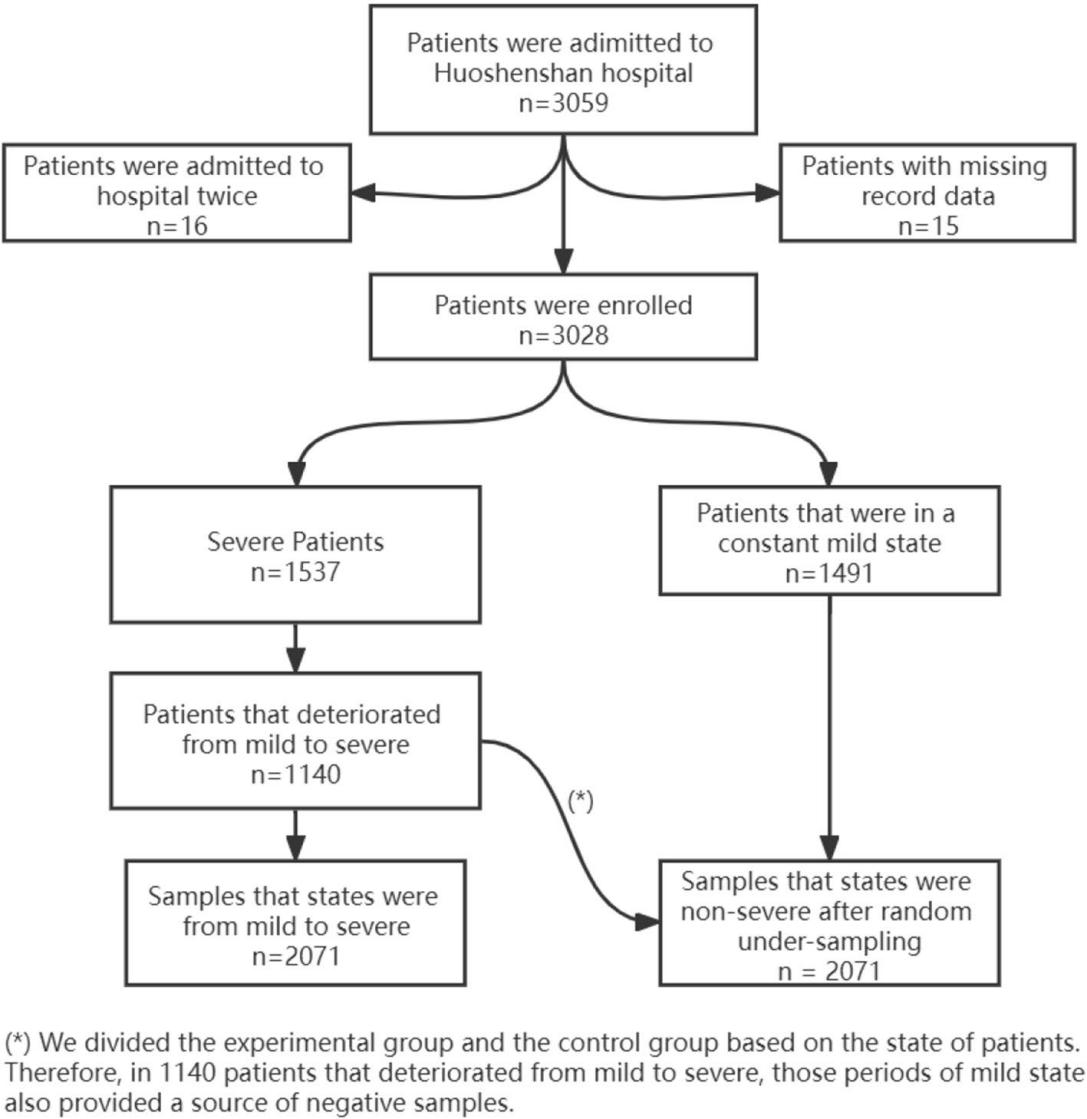
The sample set extraction process.

A total of 3028 patients were enrolled in the study, 1537 (50.8%) of whom deteriorated into severe cases (after excluding two patients with missing records). An analysis of these data revealed that 2071 mild to severe transitions occurred in 1537 patients (see Fig. 1). In this study, baseline characteristics for COVID-19 were acquired from the overall population (see Table 1) and individual samples (see Table 2). Among the entire cohort of 3,028 patients, a slight majority were male (51.1% male vs. 48.9% female). In addition, these patients generally suffered from symptoms such as fever, cough, fatigue, and dyspnea. Some patients exhibited neurological and gastrointestinal symptoms. However, compared with the patients in the non-severe group, those who deteriorated into severe cases tended to be older (median age of 63 vs. 57) and suffered from additional diseases such as hypertension (27.1% vs. 15.0%), diabetes mellitus (DM) (12.2% vs. 6.2%), coronary artery disease (CAD) (29.5% vs. 22.1%), bronchitis (5.2% vs. 2.5%), thyroid disease (12.7% vs. 8.5%), tumors (9.8% vs. 6.5%), and digestive system disease (18.5% vs 13.5%). Antihypertensive drugs were used in most patients. High doses of CCB (32.2% vs. 21.4%), ARB (6.2% vs. 2.6%), beta blockers (19.8% vs. 10.3%), and alpha blockers (2.1% vs. 0.1%) were administered to severe patients. Antibiotics were also used more commonly in severe cases, due to the presence of mixed bacterial or fungal infections in such cases (see Table 1).

**Table 1:** Baseline characteristics for COVID-19 based on population.

	Severe (n = 1537)	Non-severe (n = 1491)	*p* value
**Characteristics**			
Age (years)	63 (54.71)	57 (45.65)	< 0.01
Gender			0.814
Female	756 (49.2%)	726 (48.7%)	
Male	781 (50.8%)	765 (51.3%)	
**Comorbidities**			
Hypertension	416 (27.1%)	223 (15.0%)	< 0.01
DM	187 (12.2%)	93 (6.2%)	< 0.01
CAD	453 (29.5%)	329 (22.1%)	< 0.01
Bronchitis	80 (5.2%)	38 (2.5%)	< 0.01
Thyroid disease	195 (12.7%)	126 (8.5%)	< 0.01
Tumor	150 (9.8%)	97 (6.5%)	< 0.01
Digestive system disease	284 (18.5%)	202 (13.5%)	< 0.01
**Signs and symptoms**			
Fever	1153 (75.0%)	1069 (71.7%)	0.043
Myalgia	2 (0.1%)	2 (0.1%)	0.638
Cough	1065 (69.3%)	982 (65.9%)	0.048
Fatigue	903 (58.8%)	826 (55.4%)	0.068
Dyspnea	820 (53.4%)	698 (46.8%)	< 0.01
Oliguria	2 (0.1%)	0 (0%)	0.493
Neurological symptoms	68 (4.4%)	74 (5.0%)	0.538
Gastrointestinal symptoms	394 (25.6%)	336 (22.5%)	0.051
**Drugs**			
Antibiotics	693 (45.1%)	406 (27.2%)	< 0.01
Anti-gram-negative bacteria	662 (43.1%)	366 (24.5%)	< 0.01
Anti-gram-positive bacteria	56 (3.6%)	2 (0.1%)	< 0.01
Anti-anaerobic agents	28 (1.8%)	28 (1.9%)	0.984
Anti-atypical bacteria	43 (2.8%)	27 (1.8%)	0.092
Antifungal agents	34 (2.2%)	1 (0.1%)	< 0.01
Antihypertensive drug	669 (43.5%)	409 (27.4%)	< 0.01
CCB	495 (32.2%)	319 (21.4%)	< 0.01
ARB	96 (6.2%)	39 (2.6%)	0.061
Beta blocker	304 (19.8%)	154 (10.3%)	< 0.01
Alpha blocker	33 (2.1%)	1 (0.1%)	0.13
Hormone	371 (24.1%)	130 (8.7%)	< 0.01
Platelet aggregation inhibitors	267 (17.4%)	164 (11.0%)	< 0.01
Anticoagulants	200 (13.0%)	17 (1.1%)	< 0.01

**Table 2:** Baseline characteristics for COVID-19 based on samples.

Laboratory test	Samples from severe group (n = 2071)	Samples from non-severe group (n = 2071)	*P* value
**Vital signs**			
Heart rate (BPM)	80.00 (74.00, 86.00)	81.00 (76.00, 87.00)	< 0.01
Respiratory rate (times/min)	20.00 (18.00, 20.00)	20.00 (19.00, 20.00)	0.375
Blood oxygen saturation (%)	98.00 (97.00, 99.00)	98.00 (97.58, 99.00)	0.155
Temperature (°C)	36.50 (36.30, 36.60)	36.50 (36.30, 36.60)	0.588
**BMI**	24.29 (22.69, 25.72)	24.07 (22.35, 25.78)	0.080
**Blood routine**			
CRP (mg/L)	2.22 (0.97, 5.97)	1.53 (0.68, 4.10)	< 0.01
N%	61.30 (56.30, 68.15)	62.33 (56.40, 65.80)	< 0.01
N (10^9^/L)	3.66 (2.74, 4.49)	3.79 (2.84, 4.18)	0.841
M%	7.75 (6.70, 9.30)	7.75 (6.80, 8.80)	< 0.01
M (10^9^/L)	0.46 (0.37, 0.55)	0.47 (0.38, 0.53)	0.578
Percentage of basophil (%)	0.33 (0.20, 0.50)	0.32 (0.30, 0.50)	0.375
Basophil count (10^9^/L)	0.02 (0.01, 0.03)	0.02 (0.02, 0.03)	0.025
Percentage of eosinophil (%)	2.40 (1.30, 3.50)	2.20 (1.50, 3.10)	0.101
Eosinophil count (10^9^/L)	0.13 (0.07, 0.20)	0.14 (0.08, 0.17)	0.518
MCHC (g/L)	336.00 (330.00, 339.02)	335.00 (332.00, 339.00)	0.014
MCH (pg)	31.28 (30.30, 32.10)	31.03 (30.50, 31.80)	0.160
Nucleated erythrocyte count (/mL)	0.00 (0.00, 0.00)	0.00 (0.00, 0.00)	0.044
Percentage of nucleated erythrocyte (%)	0.00 (0.00, 0.00)	0.00 (0.00, 0.00)	< 0.01
L%	27.40 (20.60, 31.40)	27.28 (23.20, 32.30)	< 0.01
L (10^9^/L)	1.54 (1.14, 1.81)	1.67 (1.27, 1.82)	< 0.01
Leukocyte (10^9^/L)	5.90 (4.80, 6.80)	6.20 (4.90, 6.60)	0.259
Erythrocyte (10^12^/L)	3.91 (3.52, 4.17)	3.93 (3.72, 4.25)	< 0.01
RDW (%)	13.11 (12.70, 13.80)	12.90 (12.50, 13.50)	< 0.01
Hematocrit (%)	36.05 (32.90, 38.40)	36.47 (34.40, 39.10)	< 0.01
MCV (fL)	92.90 (90.55, 95.60)	92.82 (90.80, 94.70)	0.014
Mean platelet volume (fL)	10.00 (9.40, 10.70)	9.76 (9.40, 10.60)	< 0.01
Platelet count (10^9^/L)	219.00 (180.00, 256.00)	237.00 (191.00, 265.28)	< 0.01
Hemoglobin (g/L)	121.00 (109.00, 129.00)	121.72 (115.00, 132.00)	< 0.01
**Urine routine**			
Urine specific gravity			
Urine pH	5.77 (5.49, 6.08)	5.76 (5.50, 6.02)	0.723
**Coagulation**			
D-dimer (mg/L)	0.61 (0.37, 1.13)	0.39 (0.24, 0.70)	< 0.01
Prothrombin activity (%)	95.20 (93.45, 98.10)	95.20 (94.60, 97.20)	0.987
PT (s)	12.92 (12.38, 13.26)	12.91 (12.56, 13.04)	0.971
TT (s)	15.34 (14.80, 15.92)	15.28 (14.69, 15.72)	< 0.01
INR	1.08 (1.03, 1.11)	1.08 (1.05, 1.09)	0.978
APTT (s)	28.14 (26.51, 29.22)	28.57 (27.14, 29.35)	< 0.01
Fibrinogen (g/L)	3.02 (2.77, 3.39)	2.94 (2.67, 3.30)	< 0.01
**Blood biochemistry**			
α-HBDH (IU/L)	149.53 (131.95, 173.80)	148.78 (127.20, 155.42)	< 0.01
r-GT (IU/L)	32.31 (23.10, 49.72)	30.20 (21.80, 42.20)	< 0.01
LDH (IU/L)	184.30 (162.45, 211.10)	183.70 (156.45, 191.35)	< 0.01
BUN (mmol/L)	4.57 (3.83, 5.56)	4.22 (3.69, 5.11)	< 0.01
Uric acid (μmol/L)	274.00 (233.00, 325.00)	276.00 (229.31, 324.00)	0.202
Total carbon dioxide (mmol/L)	24.30 (23.20, 25.90)	24.54 (23.50, 25.50)	0.611
Bile acid (μmol/L)	4.33 (2.90, 6.80)	4.00 (2.90, 5.90)	0.019
Tbi (μmol/L)	9.50 (7.20, 11.30)	10.00 (7.60, 10.90)	0.340
Cl (mmol/L)	106.00 (104.10, 107.50)	106.36 (104.90, 107.20)	< 0.01
Globulin (g/L)	26.80 (25.12, 29.00)	26.90 (25.60, 28.50)	0.924
Alb (g/L)	36.80 (34.30, 39.00)	38.23 (35.90, 39.88)	< 0.01
dTbi (μmol/L)	3.10 (2.40, 4.00)	3.24 (2.50, 3.74)	0.435
ALP (IU/L)	70.90 (61.00, 84.00)	71.16 (60.18, 82.20)	0.118
Phosphorus (mmol/L)	1.13 (1.01, 1.25)	1.12 (1.04, 1.23)	0.544
Cr (μmol/L)	63.90 (54.59, 74.05)	63.30 (54.50, 72.92)	0.503
CK (IU/L)	40.00 (29.80, 56.20)	48.08 (34.75, 61.46)	< 0.01
CK-MB (IU/L)	8.45 (6.90, 10.20)	8.56 (7.00, 9.63)	0.139
CysC (mg/L)	0.96 (0.86, 1.10)	0.92 (0.84, 1.03)	< 0.01
Magnesium (mmol/L)	0.90 (0.86, 0.93)	0.91 (0.89, 0.93)	< 0.01
Blood glucose (mmol/L)	4.99 (4.61, 5.78)	4.84 (4.52, 5.44)	< 0.01
ALT (IU/L)	23.12 (15.90, 35.00)	24.10 (16.20, 32.50)	0.397
AST (IU/L)	19.70 (16.15, 24.60)	19.96 (16.20, 23.45)	0.066
Calcium (mmol/L)	2.17 (2.10, 2.23)	2.18 (2.13, 2.23)	< 0.01
Sodium (mmol/L)	141.03 (139.70, 142.70)	141.55 (140.60, 142.40)	< 0.01
Potassium (mmol/L)	4.27 (4.00, 4.55)	4.37 (4.10, 4.51)	< 0.01
Indirect bilirubin (μmol/L)	6.21 (4.73, 7.53)	6.73 (4.98, 7.38)	0.300

Laboratory indicators were also acquired from the sample data. The results for severe and non-severe patients differed significantly, particularly in the DD (0.61 vs. 0.39), N% (61.30 vs. 62.33), L (1.54 vs. 1.67), CRP (2.22 vs. 1.53), Alb (36.80 vs. 38.23), LDH (184.30 vs. 183.70), CK (40.00 vs. 48.08), and CysC (0.96 vs. 0.92) levels. However, there was no significant difference in the percentages of eosinophil, eosinophil count, MCH, Tbi, Cr, or ALT between the two groups of patients (see abbreviations).

### Visualization of feature importance

**Figure 2:**
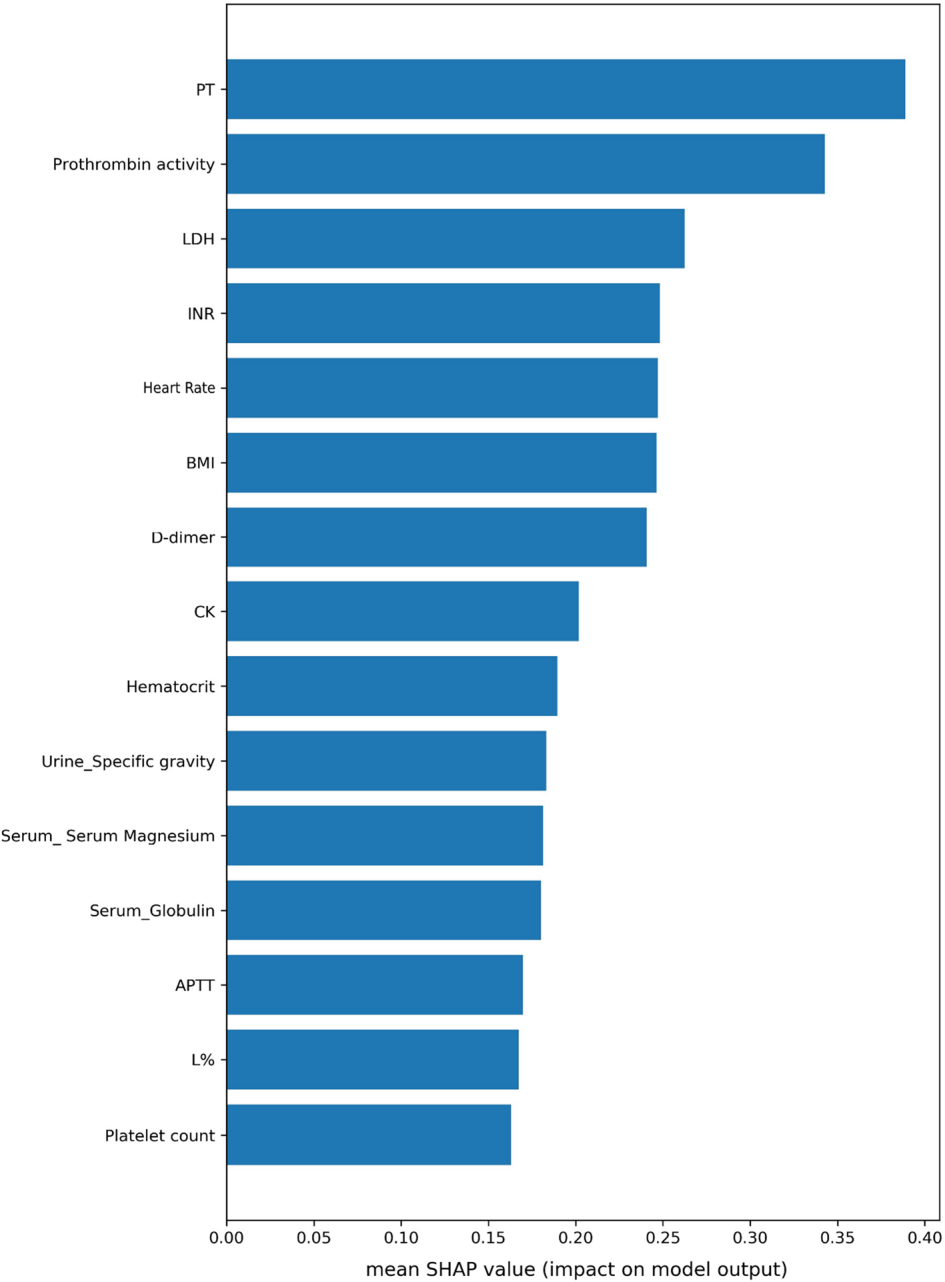
Importance rankings according to the mean absolute SHAP value. Abbreviations are as follows. PT: Prothrombin time, LDH: lactate dehydrogenase, INR: international normalized ratio, DD: D-dimer, CK: creatine kinase, APTT: activated partial thromboplastin time, L: lymphocyte count, SHAP: Shapley additive explanations.

An intuitive explanation of the importance of input model features (for clinicians) requires a ranking of features based on the XGBoost algorithm. The 15 selected features, correlating with severe COVID-19, were illustrated using a mean SHAP value plot (see Fig. 2). Among these, the top three features were PT (mean SHAP value of 0.5426), PTA (0.4450), and LDH (0.4140). In addition, a partial dependency plot was produced for each indicator, to illustrate the impact of individual metrics on the exacerbation of COVID-19. We found that lower PT, PTA, HCT, platelet count, and INR, as well as higher DD, L%, and APTT values were high-risk factors for severe COVID-19. Among the blood-based biochemical indicators, lower magnesium and globulin and higher LDH were correlated with disease deterioration. Additionally, we found that a higher BMI, a heart rate that was either too fast or too slow, and a high urine specific gravity were all risk factors for patient deterioration.

### Comparisons between XGBoost and LR

**Figure 3:**
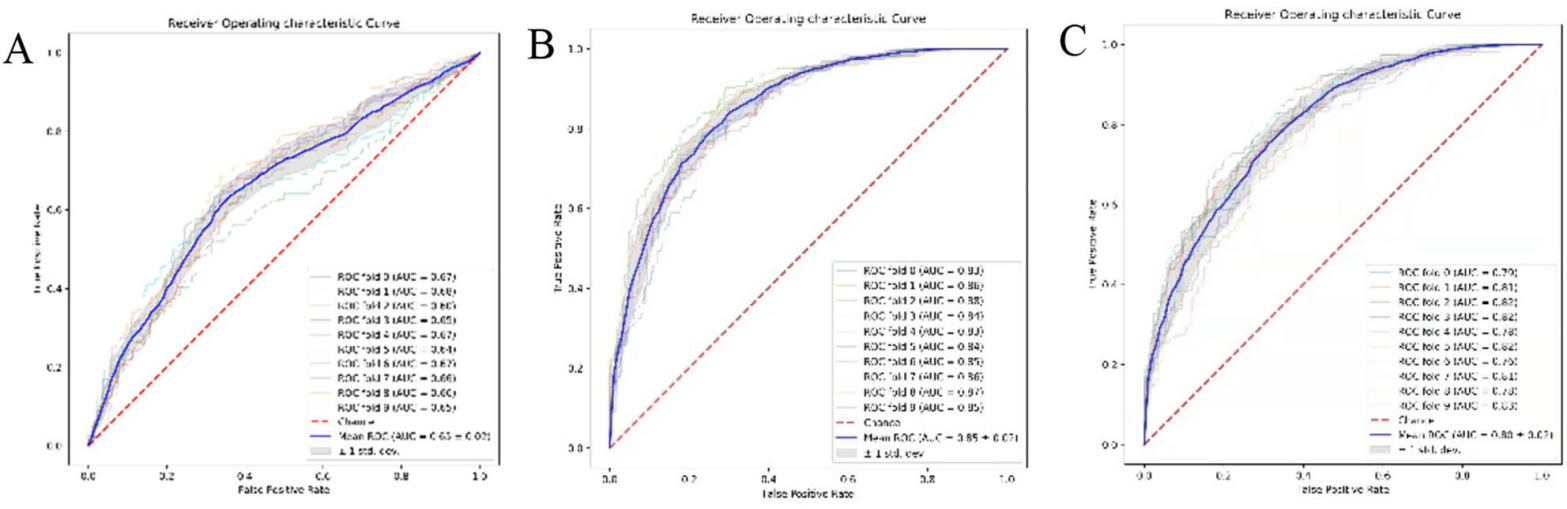
Receiver operating characteristic curves showing the performance of (**A**) LR (a combination of 15 indicators), (**B**) XGBoost (15 indicators), and (**C**) XGBoost (5 indicators) in predicting COVID-19 malignancy. AUC: area under the curve, LR: logistic regression, XGBoost: extreme gradient boosting.

The model used to predict malignant disease progression was constructed using LR and the XGBoost algorithm, respectively. XGBoost resulted in a significantly higher AUC than LR (mean AUC 0.8517, 95% CI 0.8433—0.8601 vs. AUC 0.6532, 95% CI 0.6421—0.6642, respectively; see Fig. 3). These results were used to identify optimal XGBoost parameters and rank the importance of individual features, to model the refinement metric. Detailed metrics describing the performance of these two models are provided in Table 3. Taken together, these outcomes demonstrate the value of XGBoost and SHAP plots in providing physicians with an intuitive view of key features that can accurately predict whether malignant progression will occur in mild patients.

**Table 3:** A summary of LR and XGBoost predictions.

	LR (15)	XGBoost (15)	XGBoost (5)
Accuracy	0.6272	0.7682	0.7059
Precision	0.6429	0.7652	0.6986
Recall	0.5743	0.7747	0.7251
F_1_ score	0.6058	0.7697	0.7110
AUC	0.6532	0.8517	0.7941
95% CI	(0.6421, 0.6642)	(0.8433, 0.8601)	(0.7926, 0.8151)

The number in brackets represents the number of indicators input to the model.

## Discussion

COVID-19 has been responsible for more total deaths than diseases with much higher overall case-fatality rates (e.g., SARS and MERS), due to increased transmission speed and a growing number of cases^2^. With the worldwide outbreak of COVID-19, SARS-CoV-2 infections have become a serious threat to public health. As such, early prediction and aggressive treatment of mild patients at high risk of malignant progression are critical for reducing mortality, optimizing treatment strategies, and maintaining healthcare systems^40^. This study demonstrated that a high-performing prediction model, based on XGBoost (AUC 0.8517, 95% CI 0.8433, 0.8601), could identify mild patients at risk of deteriorating into severe cases using commonly available EMR data. The proposed model also outperformed a conventional LR technique (AUC 0.6532, 95% CI 0.6421, 0.6642). Furthermore, we identified risk factors for the development of severe COVID-19 with a visual interpretation of feature importance, using SHAP plots.

**Figure 4:**
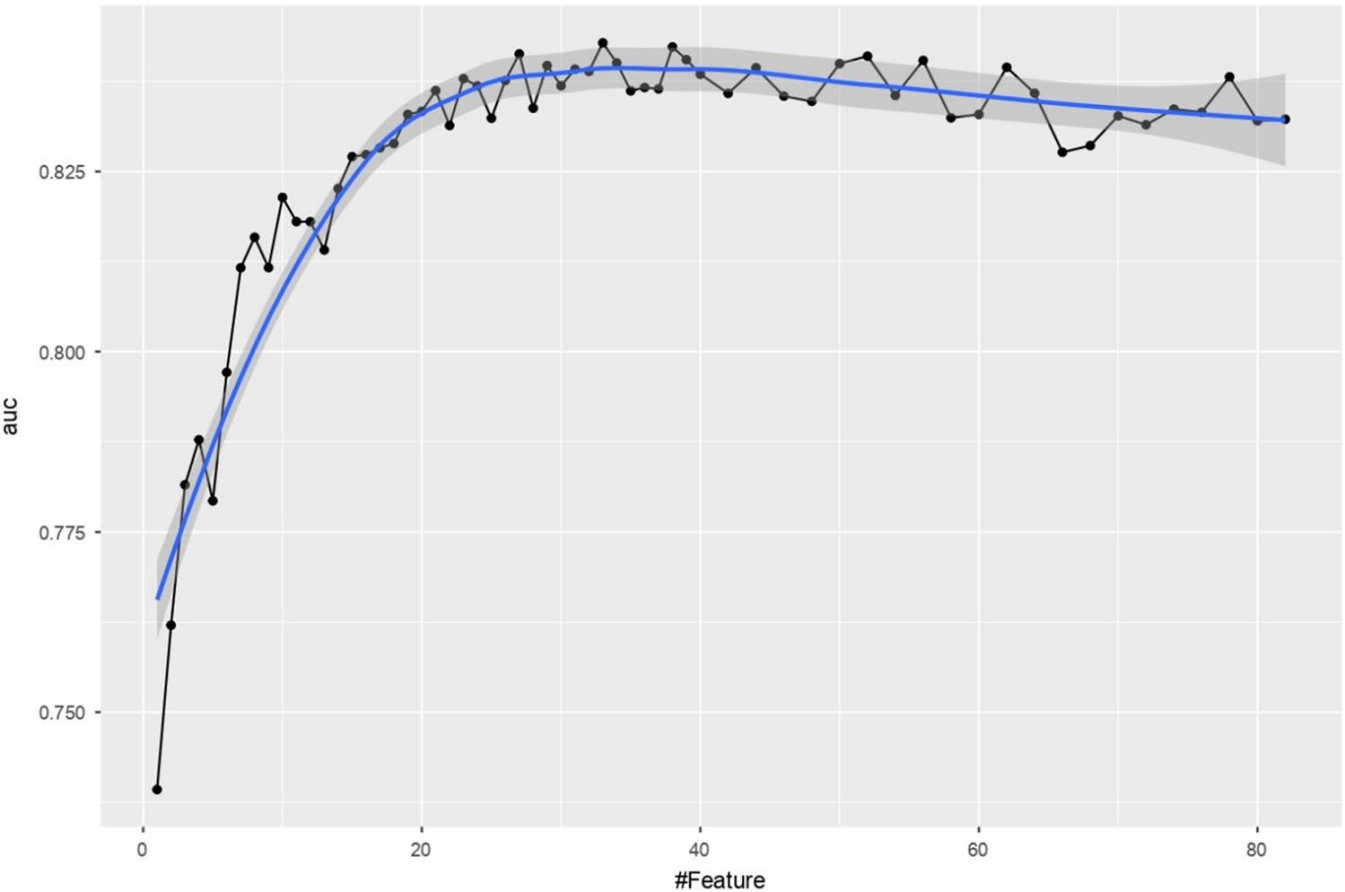
AUC for XGBoost during each iteration.

Each sample in our dataset exhibited 82 features, including comorbidities, vital signs, coagulation, blood routine, blood biochemistry, and urine routine. The set of selected indicators must then be large enough to sufficiently represent a patient’s state but not too large to be practical. This is because a patient’s condition may deteriorate while awaiting the results of laboratory tests, which affects the timeliness of diagnosis and treatment. As such, a backward stepwise method was implemented in which all features were input to the XGBoost model and their corresponding Shapely values were calculated^41^. In each iteration, the feature with the smallest absolute Shapley value was removed from the model. This process continues to iterate until no features meet the criteria for elimination and the AUC of each iterative process is recorded (see Fig. 4). The one standard error rule was used to select 15 indicators with relatively high AUC values, thus balancing efficiency requirements while maintaining prediction performance^42^. In addition, SHAP plots were utilized to explain the overall effect of XGBoost in the form of specific feature contributions, which improved the interpretability of the model (Fig. 10D).

Previous studies have focused on the use of diagnostic models for detecting COVID-19 infections, predicting mortality rates, or quantifying the risk of progression to a severe or critical state^43^. In addition, we quantified the importance of risk factors and illustrated how each factor affected the outcome. An approximate warning range was then acquired for each using partial SHAP dependency plots.

**Figure 5:**
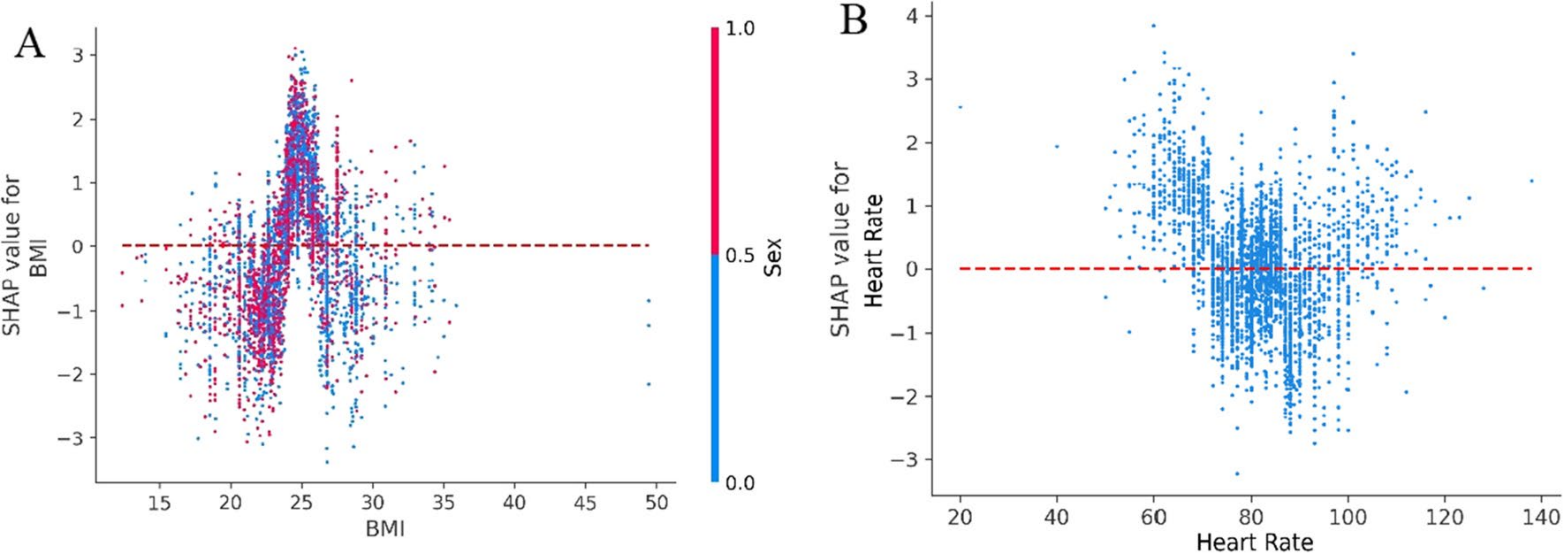
Partial SHAP dependence plots for BMI and heart rate. (**A**) BMI and gender interaction (red for male and blue for female). (**B**) Heart rate.

BMI, a commonly used international indicator to measure the degree of human obesity, has also attracted the attention of researchers in the study of risk factors for COVID-19^44–50^. These results suggest that obese patients are more likely to progress to a severe state of COVID-19^44,45^ and BMI can be used as a clinical predictor of adverse consequences^46,47,49,50^. Grigoris et al. suggested that COVID-19 patients with a BMI higher than 30 were at high risk of death^48^. The present study also found BMI to be an important risk factor affecting patient deterioration, with values in the 24–27 range representing high risk for both male and female, especially men with a BMI over 27 (Fig. 5A).

Vital signs are the most accessible indicators for patients. As such, Dara et al. developed a tool for COVID-19 risk assessment using heart rate and respiratory rate^50^. Similarly, the present study identified increased or decreased heart rate as a risk factor, reflecting the degree of dyspnea in patients. The results suggested a heart rate of less than 70 or more than 100 BPM in COVID-19 patients should be considered an early warning sign (see Fig. 5B).

**Figure 6:**
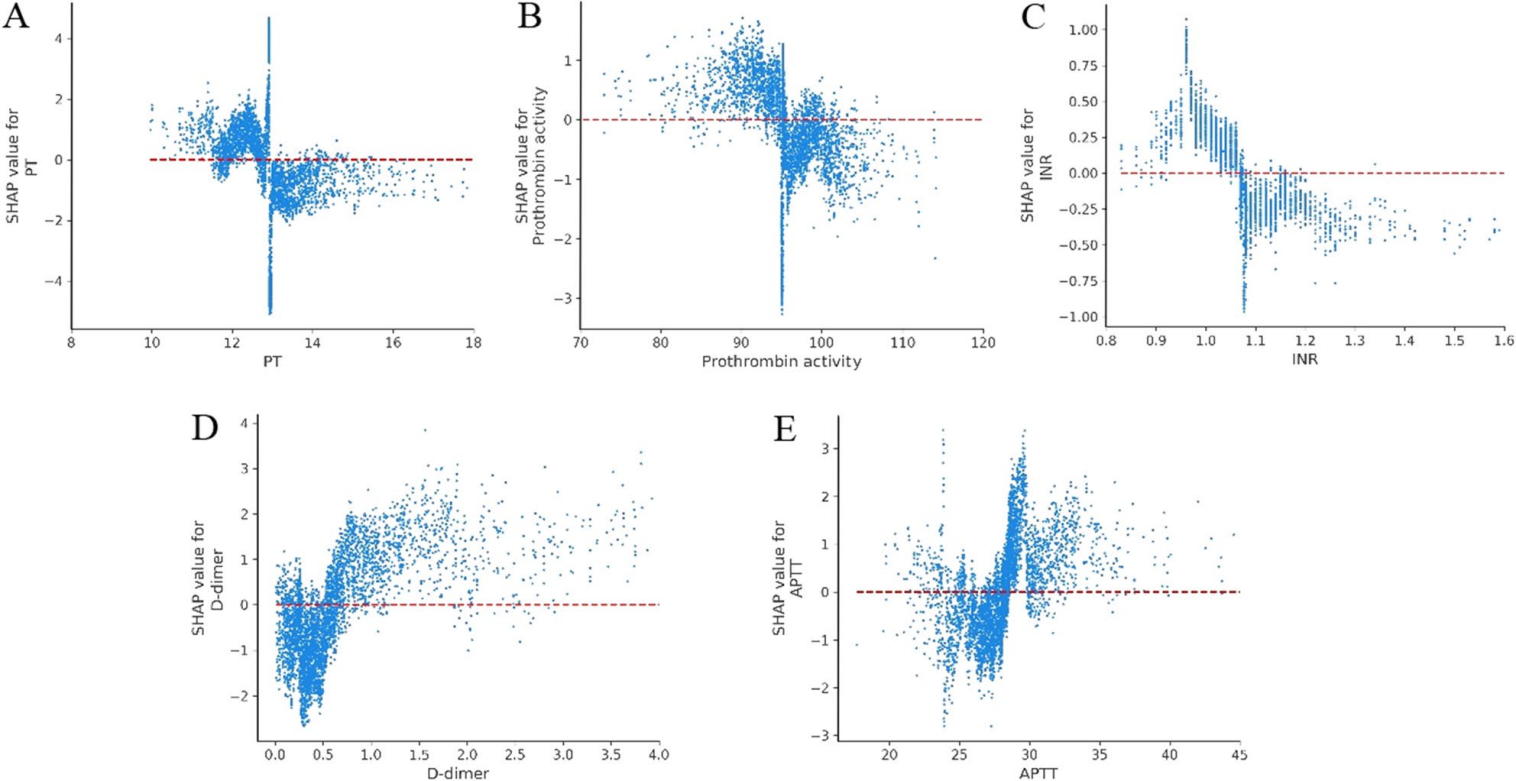
Partial SHAP dependence plots for blood coagulation. (**A**) PT. (**B**) PTA. (**C**) INR. (**D**) D-dimer. (**E**) APTT. PT: prothrombin time; PTA: prothrombin activity; INR: International normalized ratio; APTT: Activated partial thromboplastin time.

Coagulation indicators have also been shown to play a vital role in predicting the deterioration of COVID-19 patients. PT, INR, DD, and APTT have been investigated in previous studies^12–15,48,51–56^. Similarly, we identified PT, PTA, INR, DD, and APTT as risk factors and further determined their approximate warning ranges. PT was found to be the single most important indicator of malignant progression, with levels below 13 s requiring increased attention (the normal range is 11-15 s). PT values above 13 s were negatively correlated with malignant progression (see Fig. 6A). PTA was also identified as an important factor, with significant risk beginning below 96% (see Fig. 6B). In addition, SHAP values were positive for INR < 1.08 (see Fig. 6C). Previous studies have found that patients with COVID-19 are at higher risk for venous thromboembolism (VTE), which is associated with increased DD levels^53,55,56^. DD was identified as an important risk factor in this study, beginning above 0.5 mg/L (see Fig. 6D). In contrast, lower levels (DD < 0.5 mg/L) were indicative of much lower risk, with far fewer participants progressing from mild to severe status. We also found that SHAP values were positive for APTT above 28, indicating increased risk (see Fig. 6E). One of the primary contributions of this study is the first approximate early warning ranges for PT, PTA, INR, DD, and APTT levels. This could have important clinical significance for subsequent anticoagulant treatment timing and drug selection to prevent the malignant progression of COVID-19.

**Figure 7:**
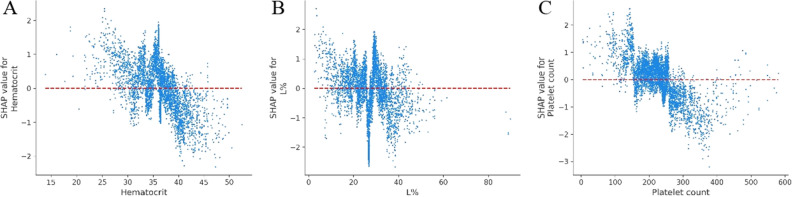
Partial SHAP dependence plots for blood routine. (**A**) Hematocrit. (**B**) L%. (**C**) Platelet count. L: Lymphocyte count.

Lymphocyte and platelet counts were also identified as biomarkers to predict patient deterioration^49,54,57^. Furthermore, L% levels above 30 and platelet counts above 280 10^9^/L were determined to be appropriate (see Fig. 7B), while a platelet count below 100 was a risk factor (see Fig. 7C). In addition, HCT values below 30 increased the risk of patient deterioration, while HCT above 40 was normal (see Fig. 7A).

To further increase clinical efficiency, we propose using only 5 indicators to predict patient deterioration. By analyzing the weights of each indicator, and incorporating recommendations from clinicians, we selected PT, heart rate, BMI, and HCT. While each of these factors ranked highly and was easily accessible in clinical practice, PT ranked first in terms of importance. In addition, PT and HCT can be analyzed immediately using POCT (point of care testing), eliminating the need for complex laboratory procedures. BMI can be calculated by simply measuring the patient's height and weight. Heart rate can also be collected quickly using a portable device that monitors vital signs. Given the impact of comorbidities on COVID-19 deterioration in clinical practice, we added comorbidity to the model as a predictor for quick pre-screening. The XGBoost algorithm was used to make predictions with only these five indicators as input, producing excellent results (AUC 0.7941, 95% CI 0.7926, 0.8151). The combination of 5 and 15 indicators can also form combined stepped indices, with different groups for varying scenarios. In addition, requiring only 5 metrics could provide rapid pre-screening, thus optimizing resource allocation.

**Figure 8:**
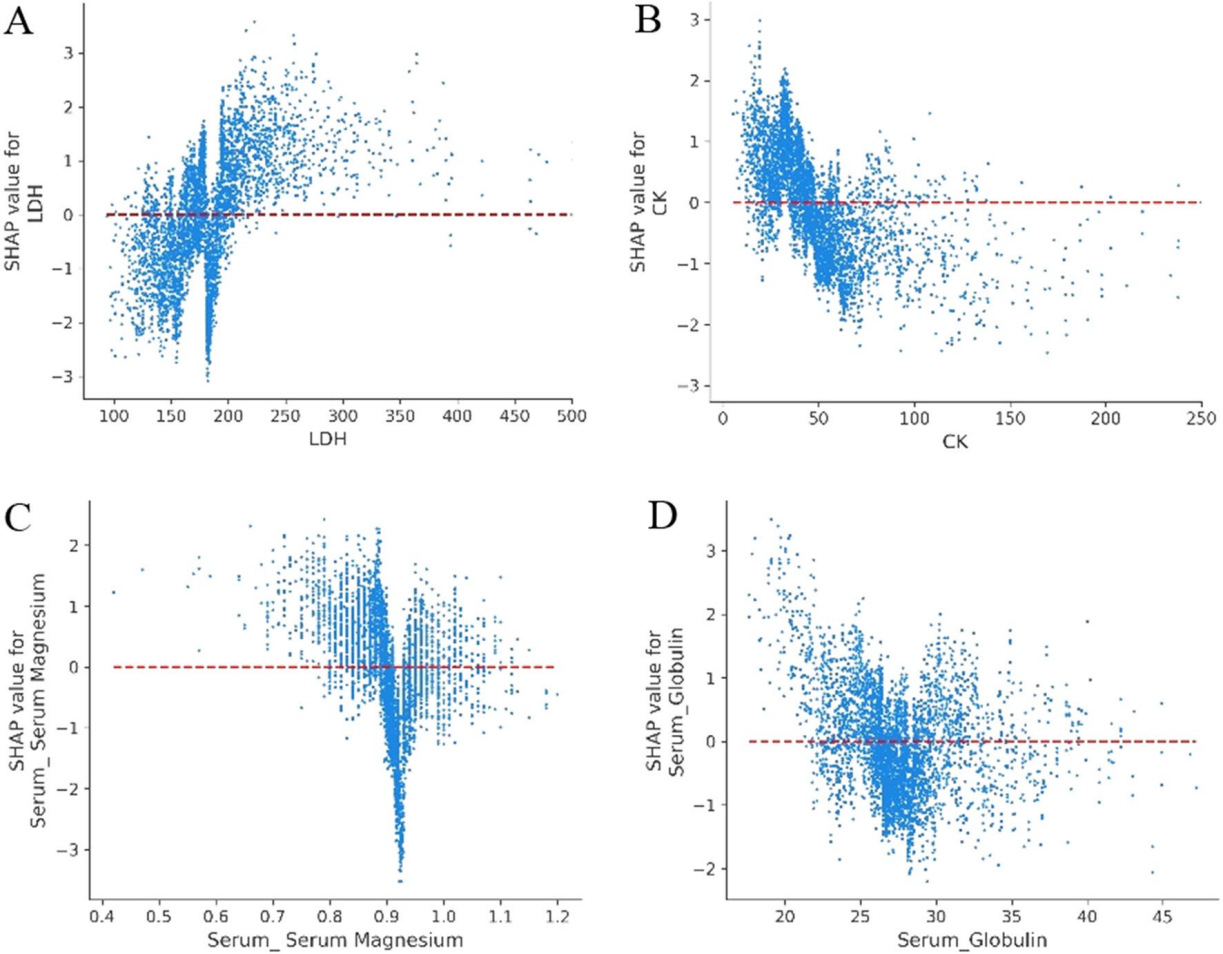
Partial SHAP dependence plots for blood biochemistry. (**A**) LDH. (**B**) CK. (**C**) Magnesium. (**D**) Globulin. LDH: lactate dehydrogenase. CK: Creatine kinase.

LDH has been shown to be predictive of poor outcomes in previous studies, such as those of Bonetti et al., Chen et al., Zheng et al., and de Terwangne et al.^13,14,51,58^ We also found LDH to be particularly useful as a risk factor at levels above 200 U/L (see Fig. 8A). Bonetti et al. and Liang et al. found that CK was associated with poor COVID-19 outcomes^13,16^. We also found CK to be a risk factor affecting patient deterioration (see Fig. 8B) and magnesium levels below 0.93 mmol/L to be a key indicator of severe COVID-19. Conversely, appropriate magnesium levels, in the range of 0.9–0.93 mmol/L, appeared to protect patients from deteriorating further (see Fig. 8C). Bonetti et al. and Albahri et al. found globulin to be a predictor of poor prognosis but did not determine corresponding early warning ranges^13,59^. We found SHAP values to be positive for globulin levels below 25 g/L (a range of 25–28 is appropriate). Globulin levels that were either too high (> 28) or too low (< 25) had an adverse effect on the development of a patient's condition (see Fig. 8D).

**Figure 9:**
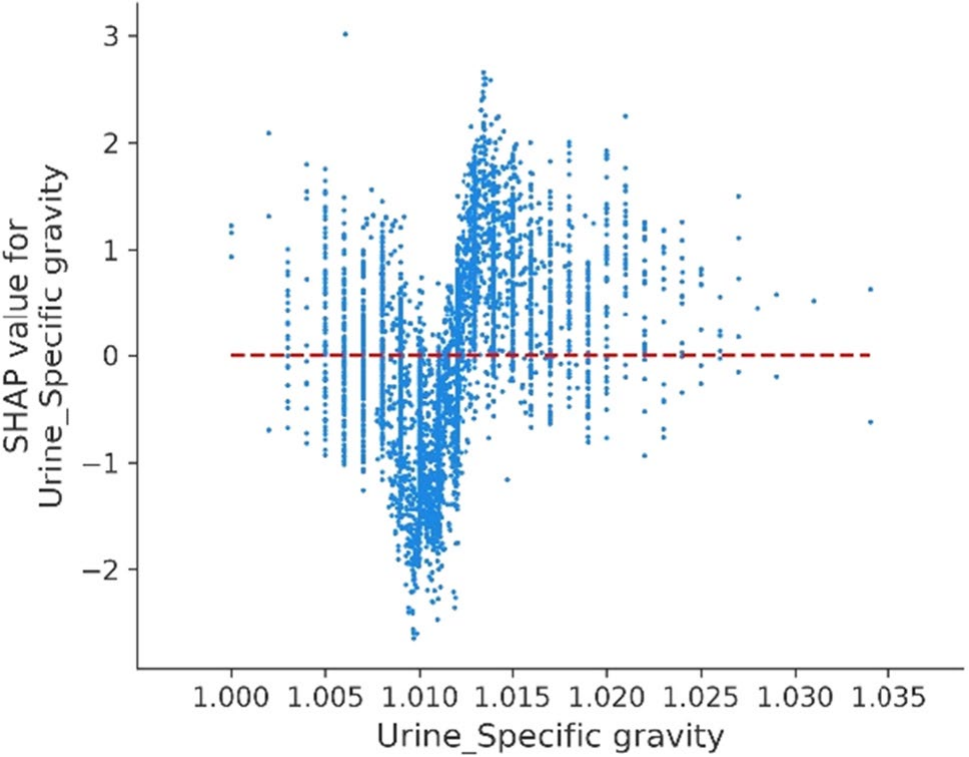
Partial SHAP dependence plots for urine routine.

Although infectious SARS-CoV-2 has been successfully isolated from urine and feces of COVID-19 patients^60–62^, studies on the variations in urine routine indicators during the deterioration of COVID-19 patients have not yet been performed. As part of this study, we first found a urine specific gravity above 1.012 to be an early warning range (Fig. 9).

This study developed a high-performing prediction model and offered valuable interpretations of quantitative findings. However, it does exhibit several inherent limitations that will need to be pursued further in a future study. For instance, the samples were analyzed retrospectively using EMR data that were not intended for the analyses performed. The Huoshenshan Hospital is a square-cabin hospital built to meet emergency needs^63^. Therefore, laboratory value indicators were not collected at regular intervals as frequently as those for critically ill patients, and data were collected at relatively long intervals. The amount of data for some of the laboratory indicators was less than that for patients in the ICU. And the impact of comorbidities on COVID-19 will be further. Although the proposed model performed well in the absence of data, the diagnosis of severe COVID-19 is a comprehensive process. As such, differences in patient profiles and healthcare could affect model performance in populations outside of China. In addition, this was a single-center study. The presence of data barriers between medical institutions in different regions prevents an external validation to verify the generalizability of the model. Finally, random under-sampling was employed to overcome the problem of class imbalances. This may have led to the discarding of potentially useful information, despite the high prediction accuracy.

### An online tool for the prediction of COVID-19 patient deterioration

**Figure 10:**
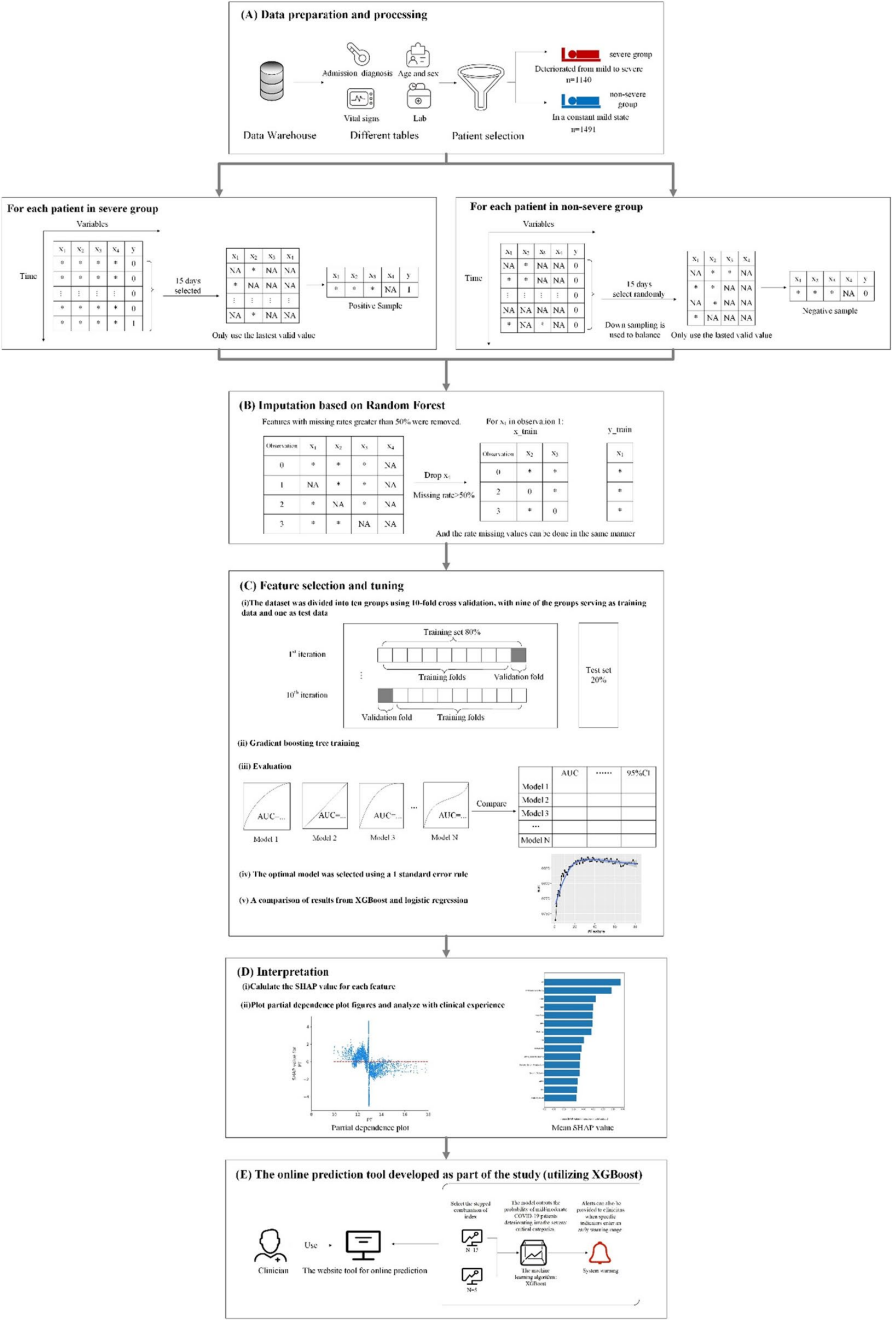
Model development overview. (**A**) Data preparation and processing. Data were extracted from a database of patients diagnosed with COVID-19, including admission diagnosis, demographic information (e.g., age and sex), vital signs, and laboratory results. Patients were divided into severe (experimental group) and non-severe (control group) categories based on whether they deteriorated into severe cases. (**B**) Imputation based on Random Forest. Features with missing rates greater than 50% were removed. (**C**) Feature selection and tuning. (i) The dataset was divided into ten groups using tenfold cross validation, with nine of the groups serving as training data and one as test data. (ii) Gradient boosting tree training. (iii) Evaluation. The AUC, F1, precision, recall, accuracy and 95% CI values were recorded and used to evaluate the performance of each model for different features and parameters. (iv) The optimal model was selected using a 1 standard error rule. (v) A comparison of results from XGBoost and logistic regression. (**D**) Interpretation. (i) The SHAP value was calculated for each feature. (ii) Partial dependence was plotted and analyzed with clinical experience. (**E**) The online prediction tool developed as part of the study (utilizing XGBoost). After selecting a combination of 15 or 4 indices, the model outputs the probability of mild/moderate COVID-19 patients deteriorating into the severe/critical categories. Alerts can also be provided to clinicians when specific indicators enter an early warning range.

Based on these findings, we have developed an online tool to predict whether the condition of patients with COVID-19 will deteriorate. The trained model is embedded at http://180.76.234.105:8001. Clinicians can select two stepped index sets based on different scenarios. When higher accuracy is required for prediction, a set of 15 indicators can be selected. When timeliness is prioritized, a set of 5 indicators can be selected. The probability of deterioration is then output by the model. In addition, if a specific indicator is in the high-risk range, it will be highlighted (Fig. 10E). This website provides a convenient and feasible means for early screening of severe patients, as well as a reference for clinicians in diagnosing patients and allocating healthcare resources.

## Conclusion

A high-performance prediction model, based on the XGBoost (AUC 0.8517, 95% CI 0.8433, 0.8601) interpretable machine-learning algorithm, was developed using EMR data from 3,028 patients. A total of 15 high-risk factors and their approximate corresponding warning ranges were identified for predicting the malignant progression of COVID-19. In addition, this study proposed the first streamlined combination of indices to achieve good predictive performance with only two laboratory indicators (PT and HCT) and two simple combinations (heart rate and BMI: AUC 0.7941, 95% CI 0.7926, 0.8151). These combined stepped indices can meet the varying needs of clinicians, providing predictive accuracy and speed for practical clinical use. A website tool was also developed for online prediction, thus improving usability and applicability. In summary, these findings could reduce mortality, improve prognosis, and optimize the clinical treatment of COVID-19 patients.

## Supplementary Information

Supplementary Information 1.

Supplementary Information 2.

Supplementary Information 3.

## Data Availability

The datasets generated and/or analyzed during the current study are not publicly available due the confidential policy of the National Health Commission of China, but are available from the corresponding author on reasonable request.

## Abbreviations

COVID: Coronavirus disease
ICU: Intensive care unit
XGBoost: Extreme gradient boosting
LR: Logistic regression
SHAPs: Shapley additive explanations
DM: Diabetes mellitus
CAD: Coronary artery heart disease
CCB: Calcium channel blockers
ARB: Angiotensin II type I receptor blockers
BMI: Body Mass Index
CRP: C-reactive protein
N: Neutrophil count
M: Monocyte count
MCHC: Mean corpuscular hemoglobin concentration
MCH: Mean corpuscular haemoglobin
L: Lymphocyte count
RDW: Red blood cell volume distribution width
MCV: Mean corpuscular volume
PT: Prothrombin time
TT: Thrombin time
INR: International normalized ratio
APTT: Activated partial thromboplastin time
α-HBDH: α-Hydroxybutyrate dehydrogenase
r-GT: R-glutamyl transpeptidase
LDH: Lactate dehydrogenase
BUN: Blood urea nitrogen
Tbi: Total bilirubin
Cl: Chlorine
Alb: Albumin
dTbi: Direct bilirubin
ALP: Alkaline phosphatase
Cr: Creatinine
CK: Creatine kinase
CK-MB: Creatine kinase isoenzyme
CysC: Cystatin C
ALT: Alanine aminotransferase
AST: Aspartate amino transferase
