# Supplementary material for: An interpretable machine learning model based on a quick pre-screening system enables accurate deterioration risk prediction for COVID-19
Source: Sci Rep. 2021 Nov 30;11:23127. doi: 10.1038/s41598-021-02370-4 (PMC8633326; doi:10.1038/s41598-021-02370-4)
Supplement: Supplementary file 1 — Supplementary Information 1. [file 41598_2021_2370_MOESM1_ESM.docx]

**Additional file 1. Definitions of clinical classifications and complications.**

| Diagnostic criteria for COVID-19^1,2^ | **Suspected Cases**  Suspected cases should be diagnosed by considering both the epidemiological histories and clinical manifestations.  *Epidemiology:*  1. Having a history of travel or residence in Wuhan and its surrounding areas or other communities with cases reported within 14 days before the patient's onset; or  2. Having a contact history with patients (a positive result of nucleic acid test for SARS-CoV-2) within 14 days of patient's onset; or  3. Having a contact history with patients with fever or respiratory symptoms from Wuhan and its surrounding areas, or the communities with cases reported within 14 days before the patient's onset; or  4. Clustering occurrence of cases.  *Clinical Manifestations:*  1. Fever and/or respiratory symptoms;  2. Having the imaging features of pneumonia described above;  3. In the early stages, a normal or decreased total white blood cell count and a decreased lymphocyte count can be found in patients who satisfy any one of the epidemiological exposure histories as well as any two of the clinical manifestations can be diagnosed as suspected cases.  Patients with no definite epidemiological history can be diagnosed only if all the three clinical manifestations are met.  **Confirmed Cases**  Suspected cases with one of the following etiological evidences can be diagnosed as confirmed cases:  1. A positive result for nucleic acid of SARS-CoV-2 by real-time fluorescence RT-PCR;  2. The virus gene sequence is highly homologous to the known SARS-CoV-2. |
| --- | --- |
| Mild cases^1,2^ | Clinical symptoms are mild, and no pneumonia manifestation can be found in imaging. |
| Moderate cases^1,2^ | Patients have symptoms like fever and respiratory tract symptoms and pneumonia manifestation can be seen in imaging. |
| Severe cases^1,2^ | Meeting any of the following:  Respiratory distress, respiratory rate of ≥ 30 breaths/minute.  Oxygen saturation ≤ 93% in a resting state.  Arterial oxygen tension (PaO2) over inspiratory oxygen fraction (FIO2) ratio ≤ 300 mmHg. Patients with > 50% lesion progression within 24-48 hours in pulmonary imaging should be treated as severe cases. |
| Critically ill cases^1,2^ | Meeting any of the following:  Respiratory failure occurs and mechanical ventilation is required. Shock occurs.  Complicated by other organ failure that requires monitoring and treatment in ICU. |

**References**

1. New coronavirus pneumonia prevention and control program (6th ed) (in Chinese). 2020 [cited ]Available from: http://www.nhc.gov.cn/yzygj/s7653p/202002/8334a8326dd94d329df351d7da8aefc2/files/b218cfeb1bc54639af227f922bf6b817.pdf

2. Chen, T. et al. Clinical characteristics of 113 deceased patients with coronavirus disease 2019: retrospective study. *BMJ-Brit. Med. J.* **368**, (2020).
