# Supplementary material for: An interpretable machine learning model based on a quick pre-screening system enables accurate deterioration risk prediction for COVID-19
Source: Sci Rep. 2021 Nov 30;11:23127. doi: 10.1038/s41598-021-02370-4 (PMC8633326; doi:10.1038/s41598-021-02370-4)
Supplement: Supplementary file 2 — Supplementary Information 2. [file 41598_2021_2370_MOESM2_ESM.docx]

**Additional file 2. All the features of the model.**

| **Classification** | **Feature** |
| --- | --- |
| Demographics | Age |
|  | Sex |
|  | BMI |
| Basic Diseases | Hyperlipidemia |
|  | Hypertension |
|  | DM |
|  | CAD |
|  | Bronchitis |
|  | Prostate disease |
|  | Thyroid disease |
|  | Tumor |
|  | Kidney disease |
|  | Digestive system diseases |
|  | Hepatitis |
|  | Enteritis |
|  | Gastritis |
|  | Cholecystitis |
|  | Pancreatitis |
|  | Nervous system disease |
|  | Hematological system disease |
| Vital Signs | Heart Rate(BPM) |
|  | Respiratory rate(times/min) |
|  | Blood oxygen saturation(%) |
|  | Temperature(℃) |
| Blood Routine | CRP (mg/L) |
|  | N% |
|  | N (10^9^/L) |
|  | M% |
|  | M (10^9^/L) |
|  | Percentage of basophil (%) |
|  | Basophil count (10^9^/L) |
|  | Percentage of eosinophil (%) |
|  | Eosinophil count (10^9^/L) |
|  | MCHC (g/L) |
|  | MCH (pg) |
|  | Nucleated erythrocyte count (10^9^/L) |
|  | Percentage of nucleated erythrocyte (%) |
|  | L% |
|  | L (10^9^/L) |
|  | Leukocyte (10^9^/L) |
|  | Erythrocyte (10^12^/L) |
|  | RDW (%) |
|  | HCT (%) |
|  | MCV (fL) |
|  | MPV (fL) |
|  | Platelet (10^9^/L) |
|  | Hemoglobin (g/L) |
| Blood Biochemistry | α-HBDH (IU/L) |
|  | r-GT (IU/L) |
|  | LDH (IU/L) |
|  | BUN (mmol/L) |
|  | Total carbon dioxide(mmol/L) |
|  | Uric acid (μmol/L) |
|  | Bile acid (μmol/L) |
|  | Tbi (μmol/L) |
|  | Globulin(g/L) |
|  | Cl (mmol/L) |
|  | Alb (g/L) |
|  | dTbi (μmol/L) |
|  | ALP (IU/L) |
|  | Phosphorus |
|  | Cr (μmol/L) |
|  | CK (IU/L) |
|  | CK-MB (IU/L) |
|  | CysC (mg/L) |
|  | Magnesium |
|  | Glucose (mmol/L) |
|  | ALT (IU/L) |
|  | AST (IU/L) |
|  | Calcium (mmol/L) |
|  | Sodium (mmol/L) |
|  | Indirect bilirubin(μmol/L) |
|  | Potassium (mmol/L) |
| Blood Coagulation | DD (mg/L) |
|  | PTA (%) |
|  | PT (s) |
|  | TT (s) |
|  | INR |
|  | APTT (s) |
|  | Fibrinogen (g/L) |
| Urine routine | Urine specific gravity |
|  | Urine pH |

BMI: Body Mass Index, DM: diabetes mellitus, CAD: coronary artery heart disease, N: neutrophil count, L: lymphocyte count, RDW: red blood cell volume distribution width, M: monocyte count, HCT: hematocrit, CRP: c-reactive protein, MCV: mean corpuscular volume, MPV：Mean platelet volume, MCH: mean corpuscular hemoglobin, MCHC: mean corpuscular hemoglobin concentration, N: neutrophil count, BUN: blood urea nitrogen, CysC: Cystatin C, Alb: albumin, Cr: creatinine, CK: creatine kinase, Tbi: total bilirubin, r-GT: r-glutamyl transpeptidase, AST: aspartate amino transferase, dTbi: direct bilirubin, Cl: chlorine, ALP: alkaline phosphatase, ALT: alanine aminotransferase, LDH: lactate dehydrogenase, CK-MB: creatine kinase isoenzyme, α-HBDH: α-hydroxybutyrate dehydrogenase, DD: D-dimer, PT: prothrombin time, PTA: Prothrombin activity, TT: thrombin time, INR: activated partial thromboplastin time, APTT: activated partial thromboplastin time.
