# Supplementary material for: An interpretable machine learning model based on a quick pre-screening system enables accurate deterioration risk prediction for COVID-19
Source: Sci Rep. 2021 Nov 30;11:23127. doi: 10.1038/s41598-021-02370-4 (PMC8633326; doi:10.1038/s41598-021-02370-4)
Supplement: Supplementary file 3 — Supplementary Information 3. [file 41598_2021_2370_MOESM3_ESM.docx]

**Additional file 3. Model evaluation metrics.**

In this study, a 10-fold cross validation was adopted to evaluate model performance. The dataset was randomly partitioned into 10 equal-sized subsamples, nine of which were used to train the model prior to validation using the remaining subsample. Accuracy, recall, and precision scores were used to assess prediction results. These metrics can be defined as follows:

,

,

,

where TP, FP, TN, and FN denote true positives, false positives, true negatives, and false negatives, respectively. Precision describes the number of positive class predictions that belong to the positive class. Recall quantifies the number of positive class predictions resulting from all positive samples in the dataset. These two metrics are expected to be close to 1. However, increasing one indicator typically causes another to decrease. The F-score combines these two indicators and provides a more realistic measure of model performance, using both precision and recall as follows:

.

Recall and precision were considered equally important in the study (i.e., β = 1). In addition, the receiver operating characteristic curve (ROC) was used to evaluate algorithm performance. It provides a comprehensive indicator representing the continuous variables of sensitivity and specificity. The AUC (area under curve) represents the probability that a predicted positive sample is ranked above a negative sample, reflecting a classifier's ability to sort samples. Values closer to 1 indicate better model performance.
